# Refining a mouse model of progressive supranuclear palsy through inoculation of human post-mortem brain-derived tau

**DOI:** 10.1186/s13104-025-07599-0

**Published:** 2026-01-06

**Authors:** S. H. Qamar, A. Mao, R. Ferry, S. Thapa, P. Singh, M. C. Tartaglia, M. S. Pollanen, A. E. Lang, H. Tanaka, I. Martinez-Valbuena, N. P. Visanji

**Affiliations:** 1 Tanz Centre for Research in Neurodegenerative Diseases, University of Toronto, 60 Leonard Avenue, Toronto, ON M5T 0S8 Canada; 2 Krembil Brain Institute, 60 Leonard Avenue, Toronto, ON Canada

**Keywords:** Progressive supranuclear palsy, Tau, Human postmortem brain, Intracerebral inoculation, Animal model, Tau pathology

## Abstract

**Objective:**

A major obstacle to developing effective therapies for Progressive Supranuclear Palsy (PSP), a uniformly fatal 4R tauopathy, is the absence of an animal model that faithfully reproduces the anatomical, cytopathological, and spatiotemporal progression of disease. Inoculation-based models, using human postmortem brain material bearing disease-specific proteopathic tau seeds, hold great translational potential for modeling tauopathies. Here we conducted key studies towards the development of an inoculation-based PSP model, using human postmortem brain to target three subcortical nuclei impacted in early disease.

**Results:**

We evaluated the impact of five different PSP brain extracts on the extent and distribution of tau pathology following inoculation into 6hTau transgenic mice expressing all six isoforms of human tau. Our findings demonstrate that 2% sarkosyl-insoluble tau successfully recapitulates core cytopathological features of PSP when introduced into disease-relevant nuclei. However, we also identify a major limitation in the restricted yield of 2% sarkosyl-insoluble tau, which significantly impedes the scalability and reproducibility of this approach. We conclude that further progress will likely require alternative strategies to generate a stable and scalable source of tau proteopathic seeds, to support a robust and reproducible inoculation-based mouse model of PSP.

**Supplementary Information:**

The online version contains supplementary material available at 10.1186/s13104-025-07599-0.

## Introduction

Progressive Supranuclear Palsy (PSP), characterized by accumulation of four repeat (4R) tau in neurons and glia in distinct brain regions [1], is a uniformly fatal neurodegenerative disease with no existing treatment. In early disease, the substantia nigra (SN) exhibits neurofibrillary tangles and threads, the globus pallidus (GP) neuronal pathology and oligodendroglial coiled bodies, and the caudate putamen (CPu) harbors pathognomonic tufted astrocytes [1]. The spatiotemporal spread of these three distinct cytopathologies throughout the brain occurs in six sequential stages [1, 2] driven by the self-propagating prion-like behaviour of misfolded tau [3]. As a primary tauopathy, with a rapid clinical progression and strong clinico-pathologic correlation, PSP has been described as a frontrunner in translational value amongst the tauopathies [4]. However, a critical barrier in the development of desperately needed treatments, is that no animal model recapitulates the anatomic and cytopathologic hallmarks, spatiotemporal spread of pathology and progressive neurodegeneration that define the disease [5]. Current animal models therefore offer poor translational value, contributing to the failure of clinical trials of potential disease modifying therapies [6].

Inoculation-based models, using human postmortem brain extracts bearing disease-specific proteopathic tau seeds, hold great translational potential for modeling tauopathies [7]. Indeed, these studies, leveraging the self-propagating ability of misfolded tau, have demonstrated the feasibly of replicating all three key PSP cytopathologies, as well as the propagation of pathology in mouse brain [8–13]. However, studies to date, employing a range of mouse lines and a variety of methods to extract tau from the human brain, have not focussed on the key subcortical nuclei affected in PSP [8–13]. As a critical step in the development of an animal model of PSP, here we compare the effects of five different tau extraction methods from human PSP brain (as well as one extraction method from human Alzheimer’s disease (AD) brain) inoculated in the SN, GP and CPu of 6hTau transgenic mice that express all six isoforms of human tau [9] (Supplementary Fig. 1).

## Methods

### Characterisation of naïve 6hTau mice

All procedures were approved by the University Health Network Animal Care Committee (protocol #6556) under the regulation of the Canadian Council on Animal Care. hT-PAC-N + / + ;E10 + 14 ± ;Mapt-/- (6htau) mice were obtained from the University of Pennsylvania and maintained as previously described [9]. N = 6 (3 males and 3 females per group), were aged to 3, 6 and 12 months old. Animals were administered sodium pentobarbital (40 mg/kg intraperitoneally). Once an appropriate surgical plane of anesthesia was achieved (as verified by a loss of toe pinch flexor response) animals were perfused transcardiacally with PBS, a terminal procedure. Following perfusion, the right hemispheres were fixed in 4% paraformaldehyde and paraffin embedded for histology. The left hemispheres were homogenized in 10% w/v PBS and centrifuged (10,000 g, 10 min, 4 °C) and the supernatants subjected to 4R-tau seed amplification assay (SAA).

### 4R-tau SAA

SAA reactions were performed as previously described, with minor modifications [14]. All reagents were purchased from Sigma. 10 μl of biological sample (1.5 μg total protein) was added to wells containing 20 μl of reaction buffer (0.1 M PB, pH 8, 0.875 M Na3Citrate, 45 μM Poly-L-glutamic acid sodium salt), 10 μl of 50 μM ThT and 10 μl of a mixture of 0.5 mg/ml of monomeric 4R-K18 and 0.25 mg/ml of monomeric 3R-K19 (rPeptide). The plate was incubated at 37 °C in a BMG FLUOstar Omega plate reader with cycles of 1 min shaking (500 rpm double orbital) and 1 min rest. ThT fluorescence (450 ± 10 nm excitation and 480 ± 10 nm emission, bottom read) were measured every 15 min for a period of 40 h.

### Immunohistochemical analysis of tau pathology

6 µm sections were deparaffinized, rehydrated and stained for tau hyperphosphorylated at Ser202/Thr205 using AT8 (MN1020, Thermo Scientific) using an automated immunostainer (Dako, Agilent) according to manufacturer’s instructions. Sections were assessed for aberrant tau cytopathologies (neuronal, oligodendroglial, astrocytic) by a neuropathologist blinded to the animals’ treatment using a 4-point semiquantitative scale was applied: 0 = none, 1 = minimal, 2 = mild, 3 = moderate and 4 = severe [15].

### Human PSP and AD case selection

Human brain material was collected with informed consent (University Health Network Research Ethics Board, Protocol 20-5258). The frontal cortex of a 73-year-old male with a clinical diagnosis of PSP for 3 years prior to death was selected. Neuropathological examination confirmed a diagnosis of PSP and excluded AD, argyrophilic grain disease, limbic-predominant age-related TDP-43 encephalopathy and Lewy body disease. A comprehensive biochemical characterisation of this case, reported in our previous study as PSP#20 [16], revealed abundant high molecular weight tau species as well as hyperphosphorylated and oligomeric tau, and high tau seeding activity as determined using a 4R-tau SAA. For AD inoculations, a 75-year male was selected, with an 11-year history of dementia and neuropathologically confirmed AD, Braak NFT stage 6 with frequent neuritic plaques.

**Preparation of human brain extracts:**

All extracts were prepared using previously published methods, for full methodological details please refer to supplemental materials.

**10% weight/volume (w/v) brain lysate**

100 mg of human brain tissue was homogenized in 1 ml PBS in a final concentration of 10% (w/v), containing protease and phosphatase inhibitors and stored at -80 °C prior to use.

**PBS soluble tau**

PBS soluble tau was prepared as previously described [16] using 50 mg of dissected human tissue.

**0.1% Sarkosyl-Insoluble (SI) tau**

0.1% SI tau was prepared as previously described [11] using 1 g of human brain tissue homogenized in 10 volumes (w/v) extraction buffer.

**1% Sarkosyl-Insoluble tau**

1% SI tau was prepared as previously described [8–13] using 2 g of human brain tissue homogenized in 9 volumes (w/v) of extraction buffer.

**2% Sarkosyl-Insoluble tau**

1% SI tau was prepared as previously described [17] using 2 g of human brain tissue homogenized in 10 volumes (w/v) suspension buffer.

### Preparation of lysates for inoculation

Total tau was quantified using an ELISA validated for the detection of all 6 isoforms of human brain tau (352–441 amino acids) (INNOTEST hTAU Ag catalog# 81580). Total Tau yield was calculated [(total tau in ng/μl) x (final volume of tau extract in μl)/ (grams of human tissue)]. Prior to injection lysates were diluted to the required concentration and sonicated (Bioruptor Pico).

### Intracerebral injection

8-week-old 6htau mice (N = 2 per group) were injected unilateraly (2.5 μl /site) using stereotaxic surgical technique at the following coordinates from bregma and skull surface (medial–lateral, anterior–posterior, dorsal–ventral in mm): CPu (+ 1.5, + 0.75, −3.0), GP (+ 1.8, −0.34, −4.0), SN (+ 1.4, −3.16, −4.6), hippocampus (+ 2.0, 2.5, −2.4) and overlying cortex (+ 2.0, −2.5, −1.4). At experimental endpoint, animals were administered sodium pentobarbital (40 mg/kg intraperitoneally). Once an appropriate surgical plane of anesthesia was achieved (as verified by a loss of toe pinch flexor response) animals were perfused transcardiacally with PBS, a terminal procedure.

### Modified Bielschowsky stain

6 µm tissue sections were deparaffinized, rehydrated and subject to the Bielschowsky Method—Sevier-Munger Modification.

## Results

### Extraction method affects the yield of tau from PSP post-mortem brain

10% w/v brain lysates, PBS soluble extracts [16] as well as 0.1%(11), 1%(9) and 2%(17) sarkosyl insoluble (SI) tau extracts were prepared from the frontal cortex of a 73-year-old male with a diagnosis of PSP (Supplementary Fig. 2). Quantification of the tau yield using an enzyme-linked immunosorbent assay (INNOTEST, Fujirebio) demonstrated that the 10% w/v brain lysate yielded the greatest amount of total tau per gram of human brain (45,720 ng/g), followed by the PBS soluble extract (14,666 ng/g). The addition of sarkosyl had a dramatic impact on the tau yield with 0.1% sarkosyl yielding 115 ng/g, 1% sarkosyl 27.8 ng/g and 2% sarkosyl 4.3 ng/g (Supplementary Fig. 1).

### 6hTau mice do not develop spontaneous tau pathology

Importantly, we demonstrate that uninoculated 6hTau mice showed no evidence of 4R-tau seeding activity (Supplementary Fig. 3a), or AT8 immunopositivity in the brain, up to 12 months of age (Supplementary Fig. 3b-f), suggesting that, under the present conditions, 6hTau mice do not spontaneously develop detectable 4R-tau seeding capacity or deposition of hyperphosphorylated tau within the first year of life.

### Inoculation with 2% SI human-brain derived PSP tau induces hyperphosphorylated tau deposits in 6hTau mice

**Fig. 1:**
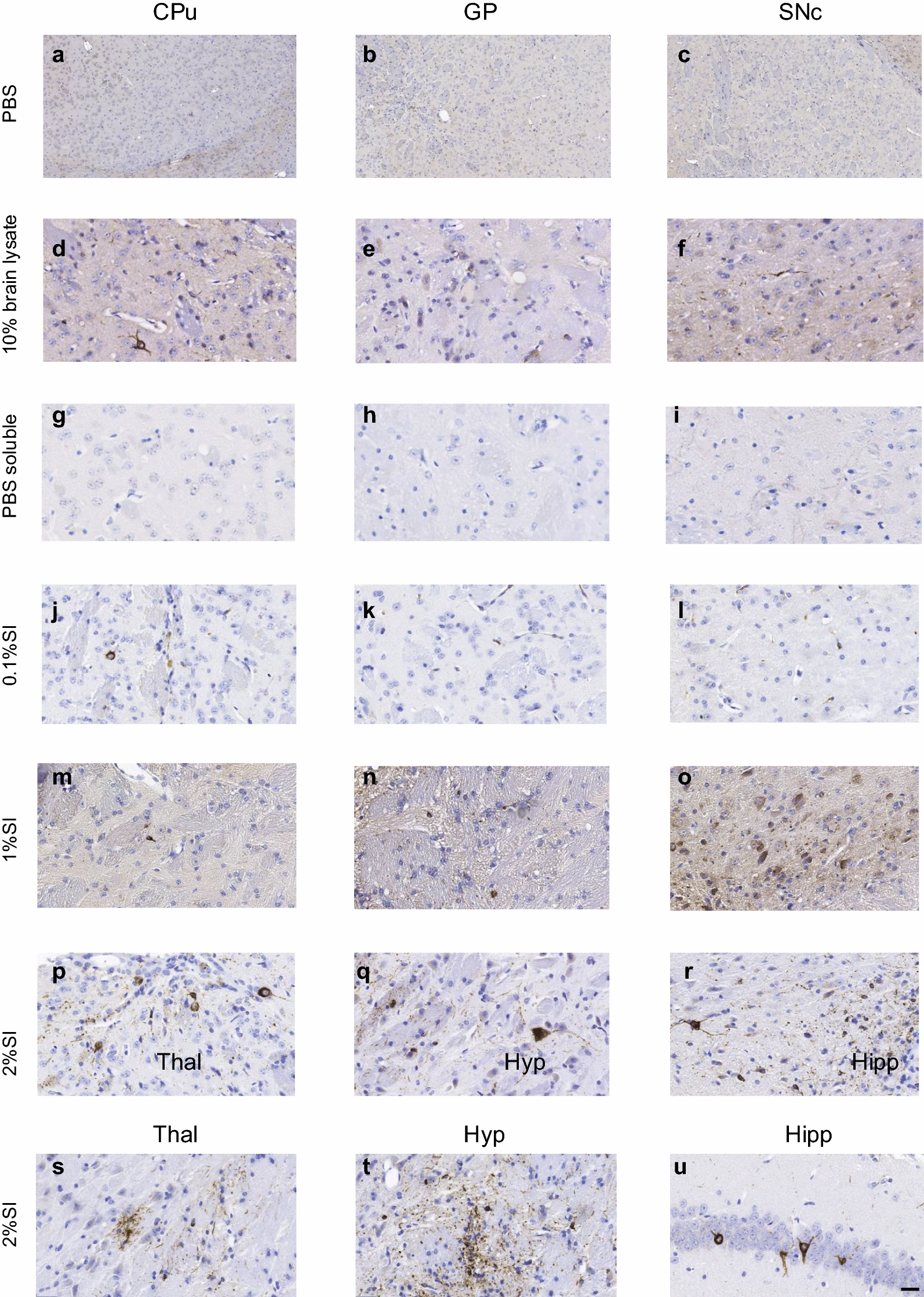
AT8 immunostaining in 6hTau mice 6 months post inoculation with PSP tau. Animals were inoculated in the caudate putamen (CPu), Globus Pallidus (GP) and Substantia Nigra pars compacta (SNc) with PBS (**a**–**c**), 10%w/v brain lysate (**d**–**f**), PBS soluble tau (**g**–**i**), 0.1% Sarkosyl Insoluble (SI) tau (**j**–**l**), 1% SI tau (**m**–**o**), 2% SI tau (**p**–**u**). Images show ipsilateral hemisphere. Scale bar shown in u represents: 100um (**a**–**c**), 20um (**d**–**u**)

Next, 3-month-old 6hTau mice were inoculated in the CPu, SN and GP with 1 ng tau per site (diluted in 2ul sterile PBS) from each of the 5 extracts, as well as PBS. Immunopositivity for hyperphosphorylated tau (AT8) was examined throughout the brain at 3- and 6-months post inoculation (mpi). Animals inoculated with PBS, as well as PBS soluble tau had little or no evidence of AT8 immunopositivity in the brain at 3 and 6 mpi (Fig. 1, Table 1). Animals inoculated with 10%w/v brain lysate, as well as 0.1% and 1% SI tau exhibited a moderate amount of AT8 immunopositivity at 6mpi with some spread to the contralateral hemisphere (Fig. 1, Table 1). In contrast, 2% SI PSP tau induced numerous AT8-positive tau deposits in neurons and neuropil threads proximal to the three inoculation sites 3 and 6 mpi, with the strongest pathology apparent in the SN. Furthermore, spread of neuronal pathology to anatomically connected regions, particularly the thalamus and hypothalamus, as well as the contralateral hemisphere was apparent at 6 mpi. (Fig. 1, Table 1). Glial pathology was scant, with only a single AT8 positive oligodendrocyte apparent in an animal inoculated with 0.1% SI tau, and a total of 3 tau positive astrocytes found across animals inoculated with 10%w/v brain lysate, 0.1% and 2% SI tau (Fig. 1, Table 1).

**Table 1:**
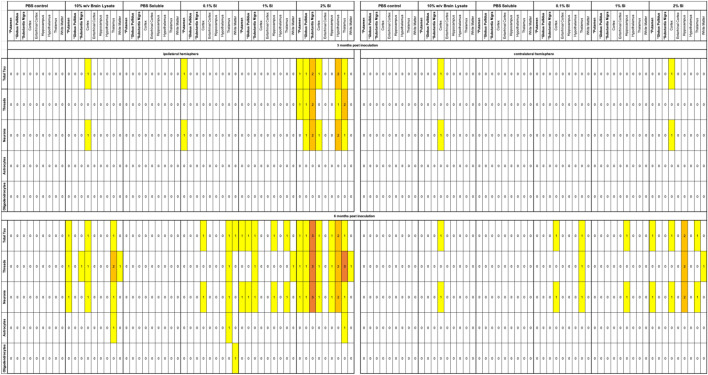
Semiquantitative Neuropathological scoring of AT8 immunopositivity in 6hTau mice 3 and 6 months post inoculation (mpi) with PSP tau

0 = none, 1 = minimal, 2 = mild, 3 = moderate, 4 = severe. * represents injection sites

### Inoculation with human-brain derived AD tau induces argentophilic tau deposits in 6hTau mice

**Fig. 2:**
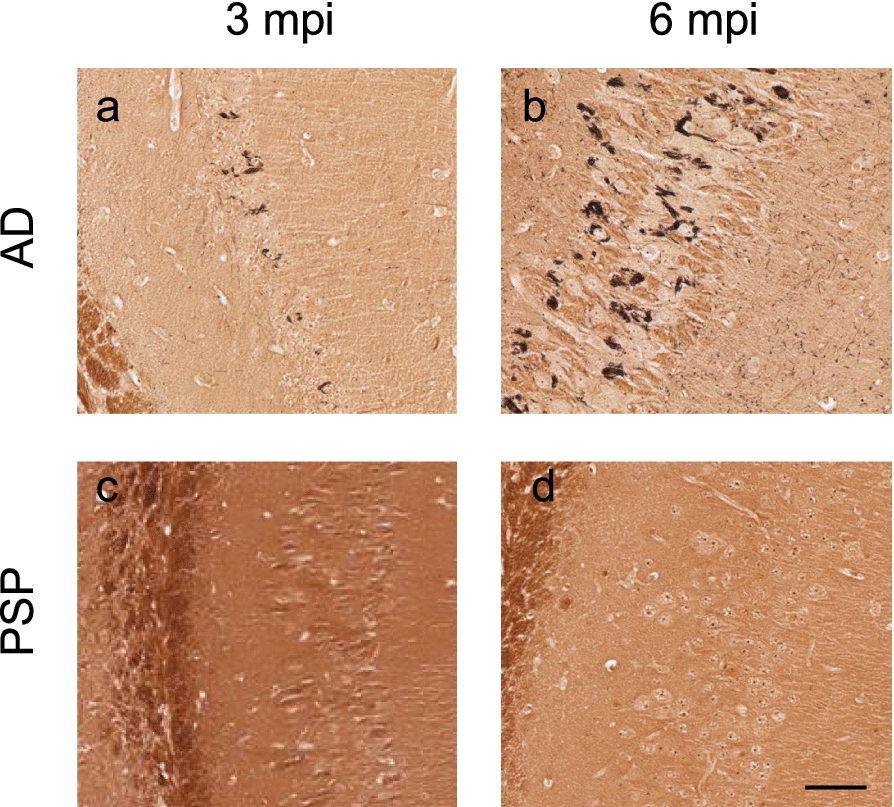
Classical neurofibrillary tangles and neuropil threads are present in AD tau- but not PSP tau-inoculated 6hTau mice. Staining with the modified Bielschowsky method at **a** 3 months post inoculation and **b** 6 months post inoculation with AD 1% SI tau in the CPu, GP and SN. Mice inoculated with PSP 2% SI tau in the CPu, GP and SN **c** 3 months post inoculation or **d** 6 months post inoculation. Scale bar, shown in d represents 50um

A separate group of 6hTau mice were inoculated in the CPu, SN and GP with 1% SI tau from a 75-year-old male with a diagnosis of AD. At 3 and 6mpi we found robust, exclusively neuronal, AT8 immunopositivity proximal to the inoculation sites with spread to several distal anatomically connected regions (Supplementary Fig. 4). Finally, we examined the argentophilic properties of tau in both PSP and AD inoculated animals. Abundant argentophilic neurofibrillary tangles and neuropil threads were observed in the hippocampus of AD tau-inoculated mice, indicating the aggregation of misfolded tau proteins in neurons and dendrites. (Fig. 2a, b). However, no silver positive neurofibrillary structures were evident in the brains of PSP inoculated animals (Fig. 2c, d).

## Discussion

Importantly, we demonstrate that 2% SI tau is the optimal extract to induce tau pathology upon inoculation, and that regions implicated in the early stages of PSP are capable of reproducing tau pathology, providing support for the use of this paradigm to model PSP. However, most tau deposits were neuronal, with only scant glial pathology apparent, and critically we did not observe the same distribution patterns of glial and neuronal pathology described in the CPu, GP and SN in PSP [1]. Monthly open-field testing showed no locomotor deficits, likely reflecting early-stage pathology (data not shown).

The yield of tau from PSP post-mortem brain decreased as increasing concentrations of sarkosyl were used, such that 1g of human PSP frontal cortex yielded sufficient 2% SI tau to induce only a modest tau pathology in the CPu, GP and SN of a single animal. Clearly this limited yield of proteopathic tau seeds from PSP post-mortem brain renders large-scale studies making direct use of patient-derived tau unfeasible. Although not explicitly stated, our findings are echoed in the literature, with studies using several grams of human brain, or pooling brain material from different patients, to obtain sufficient tau to inoculate only a modest number of animals (2–6 per study) [8–13].

Consistent with previous reports, that the yield of insoluble tau from PSP brain is lower than that of other tauopathies [12]. We found the yield of tau from AD brain was approximately double that of PSP. Furthermore, AD inoculated animals developed a robust, widespread AT8 and silver positive tau pathology, which provides a closer approximation of end-stage human pathology. Unlike AD-inoculated animals, PSP-inoculated mice failed to develop any neurofibrillary inclusions at the timepoints examined, suggesting that the deposition of hyperphosphorylated tau may occur independently of neurofibrillary tau formations and that tau hyperphosphorylation may be a preceding event to neurofibrillary tangle (NFT) development.

### Limitations

Current methods for quantifying the diverse Tau species within diseased brain tissue are inherently imperfect. Thus, when interpreting our results, consideration should be given to the possibility that the ELISA employed may fail to detect all epitopes present within a sample of aggregated Tau.

Together our findings suggest that the exciting promise of using human brain derived tau to model PSP tau pathology in animals may be significantly hampered by the small quantity of seeding competent tau that can be directly extracted from human brain. Indeed, this limits the number of animals inoculated in the present study and in published works [8–13]. This situation is further compounded by the fact that PSP is a rare and heterogenous disease, thus only limited groups have access to postmortem material from well characterized cases. For preclinical drug development, the measurement of the effect size of disease modifying therapies will likely require an animal model that develops a substantial pathological burden. Thus, to progress, the field should consider new avenues such as employing in vitro seeding amplification reactions [12], or cell-based approaches [18] to generate the *reliable, reproducible,* and *sustainable* source of PSP proteopathic seeds that are essential for the generation of a *robust, replicable* and *scalable* mouse model of PSP.

## Supplementary Information

Supplementary material 1: Figure 1: Schematic representation of PSP tau inoculation studies performed in 6htau mice. 1. The frontal cortex was obtained from a PSP postmortem brain. 2. Five different brain lysates were prepared. 3. Total tau was quantified in all lysates using an ELISA. 4. The yield of tau per gram of human brain was calculated for each of the lysates. 5. 6hTau mice were each inoculated in three subcortical nuclei implicated in early PSP. 6. Animal brains were examined for the presence of hyperphosphorylated tau (AT8 immunohistochemistry) and argentophilic neurofibrillary structures (modified Bielschowsy stain) at 3 and 6 months post inoculation. Figure 2: AT8 immunostaining in the frontal cortex of the PSP case used in the present study. AT8 immunostaining reveals abundant neuronal pathology (closed arrowhead) tufted astrocytes (open arrowhead) and oligodendroglial coiled bodies (dashed arrowhead). Scale bar 200um. Figure 3: Lack of 4R-tau seeding activity or AT8 immunopositivity in naive 6htau mouse brains at 3, 6 and 12 months of age. a) A 4R-Tau seeding amplification assay was performed in brain homogenates from a human PSP case, as well as 6hTau mice aged 3, 6 and 12 months of age. Immunostaining revealed a lack of AT8 immunopositivity throughout naïve 6hTau mouse brains at 3 (b), 6 (c) and 12 (d-f) months of age. Scale bar, show in f, represents 50um. Regions shown are caudate putamen (b-d), Globus pallidus (e) and substantia nigra pars compacta (f). Figure 4: AT8 immunostaining in 6hTau mice 6 months post inoculation with AD tau. Animals were inoculated in the caudate putamen (CPu), Substantia Nigra pars compacta (SNc) and Globus Pallidus (GP) with 1% SI tau from an AD case. Images show ipsilateral hemisphere (a-c), and both ipsilateral (right side) and contralateral hemispheres (left side) in d. Scale bar shown in d: 100um (a-c), 300um (d). Table shows semiquantitative Neuropathological scoring of AT8 immunopositivity. 0=none, 1=minimal, 2= mild, 3=moderate, 4=severe. * represents injection sites.

Supplementary Material

Table 1

## Data Availability

The datasets used and/or analysed during the current study are available from the corresponding author on reasonable request.

## Abbreviations

PSP: Progressive supranuclear palsy
4R: Four repeat
SN: Substantia nigra
GP: Globus pallidus
CPu: Caudate putamen
AD: Alzheimer’s disease
6htau: HT-PAC-N + / + ;E10 + 14 ± ;Mapt-/-
w/v: Weight/volume
SI: Sarkosyl-Insoluble
mpi: Months post inoculation
NFT: Neurofibrillary tangle
