# Supplementary material for: Refining a mouse model of progressive supranuclear palsy through inoculation of human post-mortem brain-derived tau
Source: BMC Res Notes. 2026 Jan 6;19:50. doi: 10.1186/s13104-025-07599-0 (PMC12870344; doi:10.1186/s13104-025-07599-0)
Supplement: Supplementary file 2 — Supplementary Material [file 13104_2025_7599_MOESM2_ESM.pdf]

**Preparation of Human Brain Extracts:**

**10% w/v brain lysate**

100mg of human brain tissue was homogenized in 1ml PBS in a final concentration of 10% (w/v), containing protease and phosphatase inhibitors in a gentle-MACS Octo Dissociator (Miltenyi BioTec) and stored at -80°C prior to use.

**PBS Soluble tau**

PBS soluble tau was prepared as previously described (1). 50mg of dissected human tissue was homogenized in 500µl of PBS containing protease and phosphatase inhibitors in a gentle-MACS Octo Dissociator (Miltenyi BioTec). After transferring the homogenate to a low protein binding tube, it was centrifuged at 10,000g for 10 minutes at 4°C. The supernatant was collected and stored at -80°C.

**0.1% Sarkosyl-Insoluble tau**

0.1% SI tau was prepared as previously described (2). 1g of human brain tissue was homogenized in 10 volumes (w/v) extraction buffer consisting of 10mM Tris-HCl, 0.8 M NaCl, 10% sucrose and 1 mM EGTA with protease/phosphatase inhibitors for two minutes using the gentle-MACS Octo Dissociator (Miltenyi BioTec). The homogenate was then centrifuged at 20,000 x g for 20 minutes at 4°C. Supernatant (S1) was kept, and low spin pellets were resuspended in the same buffer for centrifugation and supernatant (S2) was pooled with S1. Pellets were discarded and sarkosyl was added to the pooled supernatant to reach 0.1% (w/v) sarkosyl which was rotated for 1 hour at room temperature then centrifuged at 100,000 x g for 60 minutes at 4°C. The supernatant was discarded, and the higher spin pellets were finally resuspended in a small volume of PBS and stored at -80°C.

**1% Sarkosyl-Insoluble tau**

1% SI tau was prepared as previously described(2–7). 2g of human brain tissue was homogenized in 9 volumes (w/v) of extraction buffer consisting of 10 mM Tris-HCl, 0.8 M NaCl, Ph 7.4, 0.1% sarkosyl, 10% sucrose and 1 mM EDTA with protease/phosphatase

inhibitors for two minutes using the gentle-MACS Octo Dissociator (Miltenyi BioTec). The homogenate was then centrifuged at 10,000 x g for 10 minutes at 4°C. Supernatant (S1) was kept, and low spin pellets were subjected to two rounds of resuspension in the extraction buffer and centrifugation. Supernatants, S1, S2 and S3 were pooled together. Pellets were discarded and sarkosyl was added to the pooled supernatant to reach 1% (w/v) sarkosyl which was rotated for 1 hour at room temperature then centrifuged at 300,000 x g for 60 minutes at 4°C. Supernatant (S4) was discarded and the pellet was resuspended in PBS followed by sonication for 1 minute at 50 W using the QSonica. The sample was centrifuged at 10,000 x g for 30 minutes at 4°C. The supernatant was kept and stored at -80°C.

**2% Sarkosyl-Insoluble tau**

1% SI tau was prepared as previously described(8). 2g of human brain tissue was homogenized in 10 volumes (w/v) suspension buffer consisting of 10 mM Tris-HCl, 0.8 M NaCl, pH 7.4, 10% sucrose and 1 mM EGTA with protease/phosphatase inhibitors for two minutes using the gentle-MACS Octo Dissociator (Miltenyi BioTec). Sarkosyl was added to the homogenate to reach 2% (w/v) sarkosyl which was incubated for 30 minutes at 37°C, then centrifuged at 20,000 x g for 20 minutes at 4°C. The low spin pellet was discarded and supernatant (S1) was centrifuged at 100,000 x g for 30 minutes at 4°C. Supernatant (S2) was discarded and the pellet was resuspended in same buffer, and centrifuged at 10,000 x g for 15 minutes at 4°C. Supernatant (S3) was kept and diluted in buffer containing 0.05M Tris-HCl, 0.15 M NaCl, pH 7.4, 10% sucrose and 0.2% sarkosyl, and centrifuged at 166,000 x g for 40 minutes at 4°C. Supernatant (S4) was discarded and the pellet was resuspended in PBS and stored at -80°C.

**References for supplemental material**

1. Martinez-Valbuena I, Lee S, Santamaria E, Fernandez Irigoyen J, Li J, Tanaka H, et al. 4R-Tau seeding activity unravels molecular subtypes in patients with Progressive Supranuclear Palsy 2 3 4. Available from: https://doi.org/10.1101/2023.09.28.559953

2. Ferrer I, García MA, Carmona M, Andrés-Benito P, Torrejón-Escribano B, Garcia-Esparcia P, et al. Involvement of oligodendrocytes in tau seeding and spreading in tauopathies. Front Aging Neurosci. 2019;11(MAY).

3. Clavaguera F, Akatsu H, Fraser G, Crowther RA, Frank S, Hench J, et al. Brain homogenates from human tauopathies induce tau inclusions in mouse brain. Proc Natl Acad Sci U S A. 2013 Jun 4;110(23):9535–40.

4. He Z, McBride JD, Xu H, Changolkar L, Kim S jung, Zhang B, et al. Transmission of tauopathy strains is independent of their isoform composition. Nat Commun. 2020 Dec 1;11(1).

5. Narasimhan S, Guo JL, Changolkar L, Stieber A, McBride JD, Silva L V., et al. Pathological tau strains from human brains recapitulate the diversity of tauopathies in nontransgenic mouse brain. Journal of Neuroscience. 2017 Nov 22;37(47):11406–23.

6. Xu H, O'reilly M, Gibbons GS, Lakshmi Changolkar ·, Mcbride JD, Riddle DM, et al. In vitro amplification of pathogenic tau conserves disease-specific bioactive characteristics. Acta Neuropathol [Internet]. 2021;141:193–215. Available from: https://doi.org/10.1007/s00401-020-02253-4

7. Narasimhan S, Changolkar L, Riddle DM, Kats A, Stieber A, Weitzman SA, et al. Human tau pathology transmits glial tau aggregates in the absence of neuronal tau. Journal of Experimental Medicine. 2020 Feb 3;217(2).

8. Shi Y, Zhang W, Yang Y, Murzin AG, Falcon B, Kotecha A, et al. Structure-based classification of tauopathies. Nature. 2021 Oct 14;598(7880):359–63.

### 1. PSP Post-mortem Brain

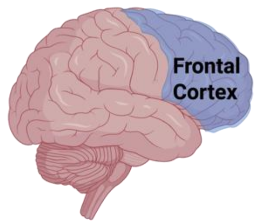


### 2.Inoculum Preparation

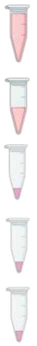

- 10% w/v brain lysate
- PBS soluble
- 0.1% sarkosyl insoluble
- 1% sarkosyl insoluble
- 2% sarkosyl insoluble

### 3.Quantification of tau ( Human tau ELISA, INNOTEST Fujiribio)

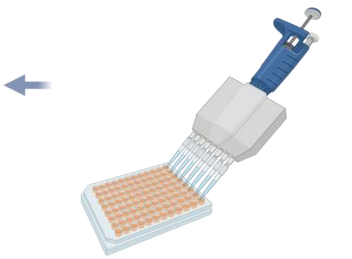

### 4. Yield of tau per unit weight of human post mortem brain

| Extraction Method | Weight of human post-mortem brain (g) | Final volume of tau extract (μL) | Total Tau (ng/(μL) | Tau Yield (ng) per gram of human brain |
|---|---|---|---|---|
| 10% w/v brain lysate: PSP | 0.1 | 900 | 5.08 | 45,720 |
| PBS Soluble: PSP | 0.05 | 500 | 1.32 | 14,666 |
| 0.1% SI: PSP | 1 | 50 | 2.28 | 115 |
| 1% SI: PSP | 1 | 25 | 1.33 | 27.8 |
| 2% SI: PSP | 2 | 25 | 0.35 | 4.3 |
| 1% SI: AD | 2 | 50 | 1.91 | 47.9 |

### 5. Stereotaxic Inoculation in 8-12 week old 6hTau mice

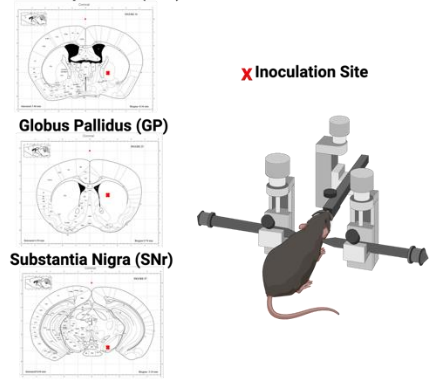


### 6. Immunohistochemistry and semiquantitative scoring of tau deposition 3 and 6 mpi

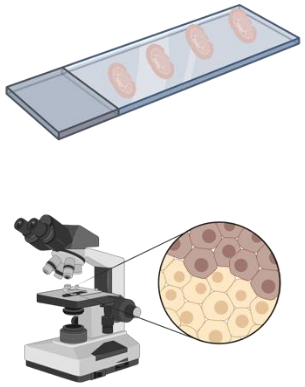

**Supplementary Figure 1: Schematic representation of PSP tau inoculation studies performed in 6htau mice.** 1. The frontal cortex was obtained from a PSP postmortem brain. 2. Five different brain lysates were prepared. 3. Total tau was quantified in all lysates using an ELISA. 4. The yield of tau per gram of human brain was calculated for each of the lysates. 5. 6hTau mice were each inoculated in three subcortical nuclei implicated in early PSP. 6. Animal brains were examined for the presence of hyperphosphorylated tau (AT8 immunohistochemistry) and argentophilic neurofibrillary structures (modified Bielschowsy stain) at 3 and 6 months post inoculation.

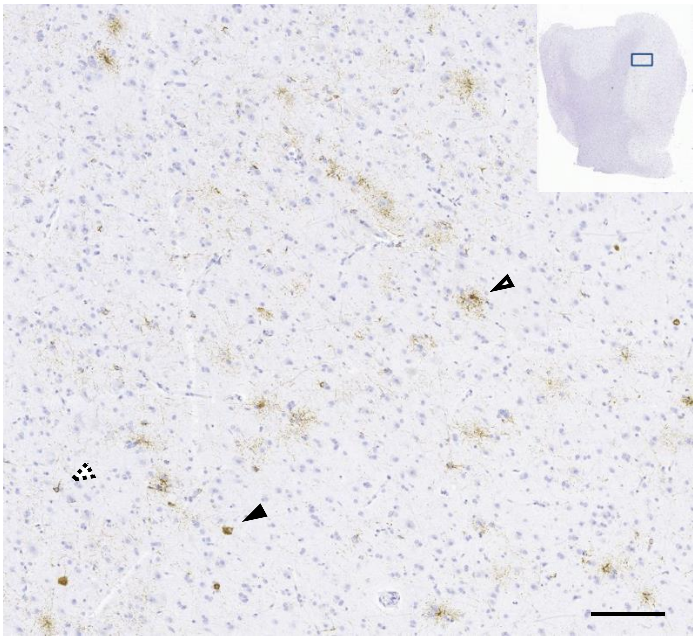

**Supplementary Figure 2: AT8 immunostaining in the frontal cortex of the PSP case used in the present study.** AT8 immunostaining reveals abundant neuronal pathology (closed arrowhead) tufted astrocytes (open arrowhead) and oligodendroglial coiled bodies (dashed arrowhead). Scale bar 200um.

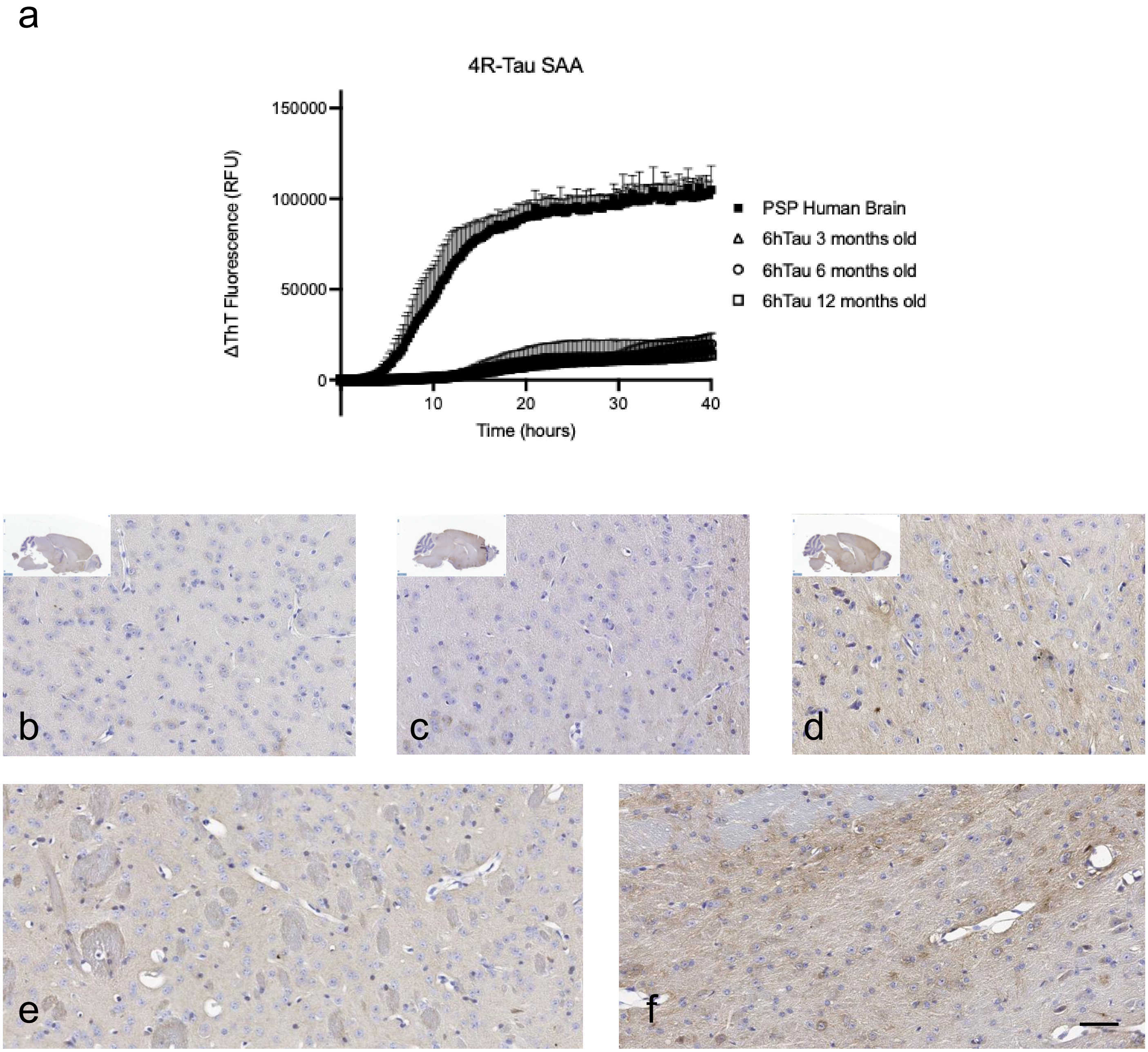


**Supplementary Figure 3: Lack of 4R tau seeding activity or AT8 immunopositivity in naive 6htau mouse brains at 3, 6 and 12 months of age.** a) A 4R-Tau seeding amplification assay was performed in brain homogenates from a human PSP case, as well as 6hTau mice aged 3, 6 and 12 months of age. Immunostaining revealed a lack of AT8 immunopositivity throughout naïve 6hTau mouse brains at 3 (b), 6 (c) and 12 (d-f) months of age. Scale bar, show in f, represents 50um. Regions shown are caudate putamen (b-d), Globus pallidus (e) and substantia nigra pars compacta (f).

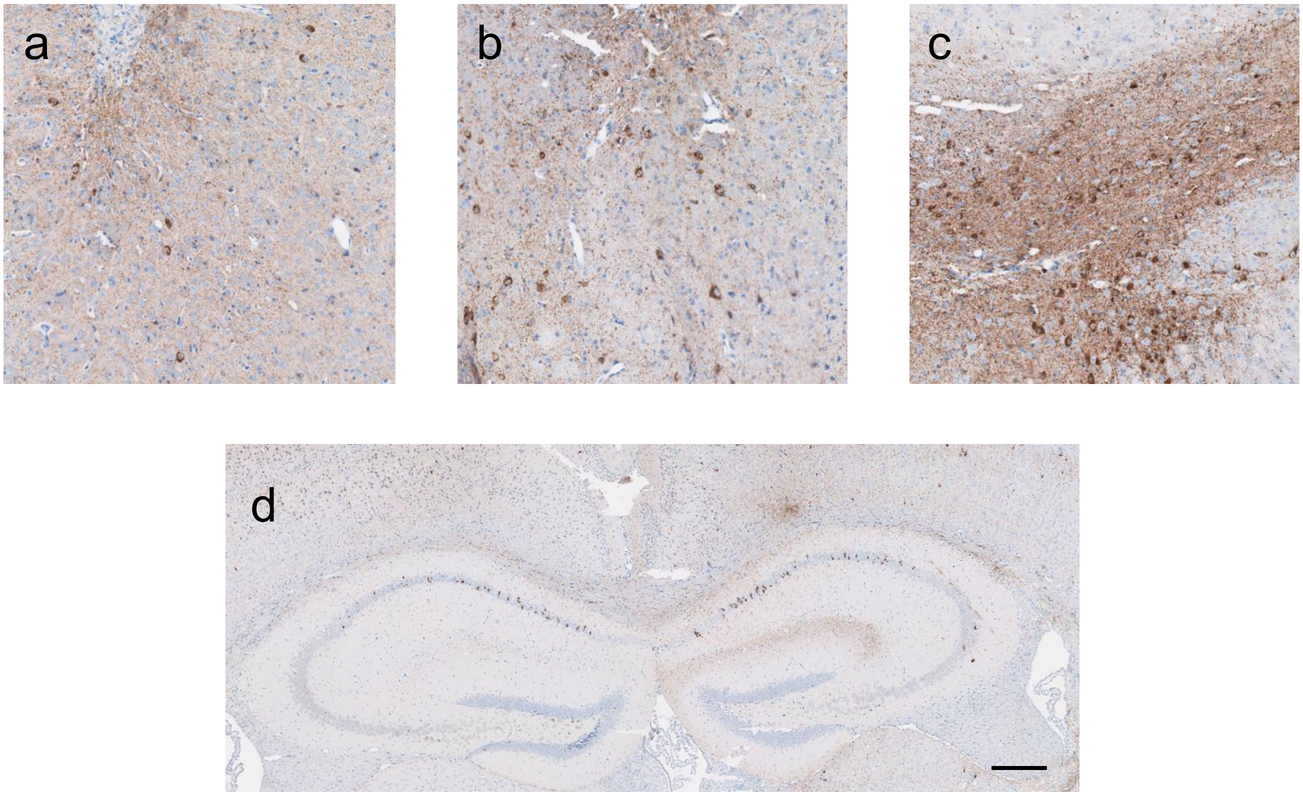


| 3 months post inoculation | ipsilateral hemisphere: *Putamen | *Globus Pallidus | *Substantia Nigra | Cortex | Entorhinal Cortex | Hippocampus | Hypothalamus | Thalamus | White Matter | contralateral hemisphere: *Putamen | *Globus Pallidus | *Substantia Nigra | Cortex | Entorhinal Cortex | Hippocampus | Hypothalamus | Thalamus | White Matter |
|---|---|---|---|---|---|---|---|---|---|---|---|---|---|---|---|---|---|---|
| Total Tau | 2 | 3 | 2 | 4 | 4 | 3 | 4 | 3 | 0 | 0 | 0 | 0 | 4 | 2 | 2 | 2 | 0 | 0 |
| Threads | 3 | 4 | 3 | 4 | 3 | 2 | 4 | 2 | 3 | 2 | 1 | 0 | 3 | 2 | 2 | 1 | 1 | 2 |
| Neurons | 2 | 3 | 2 | 4 | 4 | 3 | 4 | 3 | 0 | 0 | 0 | 0 | 4 | 2 | 2 | 2 | 0 | 0 |
| Astrocytes | 0 | 0 | 0 | 0 | 0 | 0 | 0 | 0 | 0 | 0 | 0 | 0 | 0 | 0 | 0 | 0 | 0 | 0 |
| Oligodendrocytes | 0 | 0 | 0 | 0 | 0 | 0 | 0 | 0 | 0 | 0 | 0 | 0 | 0 | 0 | 0 | 0 | 0 | 0 |

| 6 months post inoculation | ipsilateral hemisphere: *Putamen | *Globus Pallidus | *Substantia Nigra | Cortex | Entorhinal Cortex | Hippocampus | Hypothalamus | Thalamus | White Matter | contralateral hemisphere: *Putamen | *Globus Pallidus | *Substantia Nigra | Cortex | Entorhinal Cortex | Hippocampus | Hypothalamus | Thalamus | White Matter |
|---|---|---|---|---|---|---|---|---|---|---|---|---|---|---|---|---|---|---|
| Total Tau | 2 | 3 | 3 | 4 | 2 | 2 | 0 | 1 | 0 | 0 | 0 | 2 | 2 | 2 | 1 | 0 | 1 | 0 |
| Threads | 3 | 4 | 4 | 4 | 2 | 2 | 2 | 2 | 4 | 2 | 3 | 2 | 2 | 2 | 1 | 2 | 1 | 3 |
| Neurons | 2 | 3 | 3 | 4 | 2 | 2 | 0 | 1 | 0 | 0 | 0 | 2 | 2 | 2 | 1 | 0 | 1 | 0 |
| Astrocytes | 0 | 0 | 0 | 0 | 0 | 0 | 0 | 0 | 0 | 0 | 0 | 0 | 0 | 0 | 0 | 0 | 0 | 0 |
| Oligodendrocytes | 0 | 0 | 0 | 0 | 0 | 0 | 0 | 0 | 0 | 0 | 0 | 0 | 0 | 0 | 0 | 0 | 0 | 0 |

**Supplementary Figure 4: AT8 immunostaining in 6hTau mice 6 months post inoculation with AD tau.** Animals were inoculated in the caudate putamen (CPu), Substantia Nigra pars compacta (SNc) and Globus Pallidus (GP) with 1% SI tau from an AD case. Images show ipsilateral hemisphere (a-c), and both ipsilateral (right side) and contralateral hemispheres (left side) in d. Scale bar shown in d: 100um (a-c), 300um (d). Table shows semiquantitative Neuropathological scoring of AT8 immunopositivity. 0=none, 1=minimal, 2= mild, 3=moderate, 4=severe. * represents injection sites.
